# Current Trends in Neurodegeneration: Cross Talks between Oxidative Stress, Cell Death, and Inflammation

**DOI:** 10.3390/ijms22147432

**Published:** 2021-07-11

**Authors:** Tapan Behl, Rashita Makkar, Aayush Sehgal, Sukhbir Singh, Neelam Sharma, Gokhan Zengin, Simona Bungau, Felicia Liana Andronie-Cioara, Mihai Alexandru Munteanu, Mihaela Cristina Brisc, Diana Uivarosan, Ciprian Brisc

**Affiliations:** 1 Chitkara College of Pharmacy, Chitkara University, Punjab 140401, India; tapanbehl31@gmail.com (T.B.); rashitamakker32@gmail.com (R.M.); aayushsehgal00@gmail.com (A.S.); sukhbir.singh@chitkara.edu.in (S.S.); neelam.mdu@gmail.com (N.S.); 2 Department of Biology, Faculty of Science, Selcuk University Campus, Konya 42130, Turkey; biyologzengin@gmail.com; 3 Department of Pharmacy, Faculty of Medicine and Pharmacy, University of Oradea, 410028 Oradea, Romania; 4 Doctoral School of Biological and Biomedical Sciences, University of Oradea, 410073 Oradea, Romania; 5 Department of Psycho-Neuroscience and Recovery, Faculty of Medicine and Pharmacy, University of Oradea, 410073 Oradea, Romania; 6 Department of Medical Disciplines, Faculty of Medicine and Pharmacy, University of Oradea, 410073 Oradea, Romania; mihaimunteanual@yahoo.com (M.A.M.); briscristina@yahoo.com (M.C.B.); brisciprian@gmail.com (C.B.); 7 Department of Preclinical Disciplines, Faculty of Medicine and Pharmacy, University of Oradea, 410073 Oradea, Romania; diana.uivarosan@gmail.com

**Keywords:** neurodegeneration, neuroinflammation, apoptosis, necrosis, cell death, oxidative stress

## Abstract

The human body is highly complex and comprises a variety of living cells and extracellular material, which forms tissues, organs, and organ systems. Human cells tend to turn over readily to maintain homeostasis in tissues. However, postmitotic nerve cells exceptionally have an ability to regenerate and be sustained for the entire life of an individual, to safeguard the physiological functioning of the central nervous system. For efficient functioning of the CNS, neuronal death is essential, but extreme loss of neurons diminishes the functioning of the nervous system and leads to the onset of neurodegenerative diseases. Neurodegenerative diseases range from acute to chronic severe life-altering conditions like Parkinson’s disease and Alzheimer’s disease. Millions of individuals worldwide are suffering from neurodegenerative disorders with little or negligible treatment available, thereby leading to a decline in their quality of life. Neuropathological studies have identified a series of factors that explain the etiology of neuronal degradation and its progression in neurodegenerative disease. The onset of neurological diseases depends on a combination of factors that causes a disruption of neurons, such as environmental, biological, physiological, and genetic factors. The current review highlights some of the major pathological factors responsible for neuronal degradation, such as oxidative stress, cell death, and neuroinflammation. All these factors have been described in detail to enhance the understanding of their mechanisms and target them for disease management.

## 1. Introduction

The human brain is the most complex organ, controlling all the amazing things we do by regulating several molecular pathways. It comprises billions of cells, called neurons, which control the proper functioning of our body. Neurons stimulate and transmit signals that enable us to talk, move, think, and accomplish everything we do. The brain cells are closely interconnected with each other. Therefore, the slightest miscommunications within cells in a particular area can lead to a disruption in other activities controlled by brain, causing major brain disorders. Neurodegenerative diseases can also be characterized by progressive loss in the functioning of the brain due to an accumulation of toxic proteins that exhibit clinical syndromes [1,2]. Therefore, brain disorders should not be taken lightly, as they can result in widespread problems [3], ultimately leading to neuronal death and shrinkage. The word “neurodegenerative” is formed of two parts—“neuro,” which means brain, and “degenerative,” which means dying or breaking down. Inadequate communication among brain cells lead to devastating effects, influencing several activities of an individual such as movement, memory, speech intelligence, and many more [4,5]. Neurodegenerative diseases are highly complex, and their etiology is sometimes very hard to predict.

Different areas of the brain encounter different types of neurodegenerative diseases, and they are described in Table 1. Examples of neurodegenerative diseases include Parkinson’s disease, Huntington disease, Alzheimer’s disease, amyotrophic lateral sclerosis, and many more.

The symptoms of neurodegenerative diseases are mainly encountered in older groups of people [6]. This group of people is highly vulnerable to memory loss, which results in a poor quality of life and loss of personality [7,8,9]. With the increasing population worldwide, the incidence of neurological diseases is also increasing. According to published data, it has been determined that new cases of Parkinson’s disease and Alzheimer’s disease have increased abruptly over the span of the last 30 years. Around the globe, more than 10 million people are suffering from Parkinson’s disease and more than 5.4 million people are living with Alzheimer’s disease, indicating that neurodegenerative diseases are the leading cause of death worldwide and are highly prevalent in populations of people 60 years of age [10]. The massive increase in neurodegenerative diseases can be contributing to an increase in the prevalence of amyotrophic lateral sclerosis (ALS) disease. In addition, it has been determined that a large population of the elderly age group is estimated to be suffering from Huntington disease.

The prevalence of neurodegenerative diseases among all the genders, races, and geographical areas is increasing with increasing population all over the world [11]. These diseases are highly complex and difficult to cure; therefore, it has become necessary to develop newer medications with effective therapeutic strategies to overcome them. Simulated models comprising everything from unicellular organisms to the most complex functioning have been developed, and have proven to be useful tools in the research and development of new therapeutic medications by exploring the underlined advanced neurological pathways [12]. The collection of biomarkers or therapeutic targets provides greater insight to the pathophysiology of neurological diseases and can also contribute to researching new medications [13,14].

**Figure 1:**
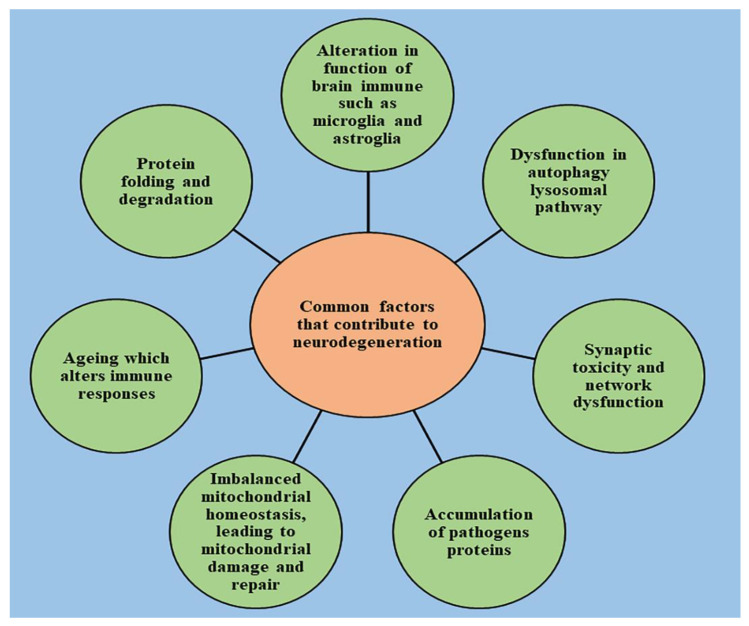
Some of the common factors responsible for the initiation/progression of neurological diseases.

Figure 1 describes some of the common factors that are responsible for the initiation and progression of neurological diseases and provide greater insight into the pathophysiology.

The current review explains the most common pathways that are responsible for the initiation and progression of commonly occurring neurodegenerative disorders.

## 2. Role of Cell Death in the Onset of Neurodegeneration

Neurological disorders are mainly characterized by increased degradation in the functioning of neurons due to the destruction of synapses and axons, eventually leading to nerve cell death. An understanding of the mechanism that leads to the homeostasis of cellular elements and neurodegradation is highly important for developing novel therapeutic treatments for the diseases [15,16,17,18,19]. The healthy cells in the human body transform to preserve the normal homeostasis of tissues; however, post-mitotic neurons harbor very little capacity to regenerate and their survival is essential to ensure the proper functioning of the nervous system [20]. The death of neurons promotes the development of nervous system; however, if occurring in excess, it leads to declined functioning of nervous system and causes the progression of neurodegenerative diseases, which can be indicated by a range of acute insults, from stroke and traumatic brain injury (TBI) to enduring critical conditions such as Parkinson’s disease [21], Alzheimer’s disease [22], and amyotrophic lateral sclerosis [23].

Several studies have been conducted to understand the neuropathology behind the chronic conditions of these diseases, and stereotypical patterns of neurodegeneration have been identified in different regions of the central nervous system, which correspond to the disease severity clinically [24]. Several other pathological pathways, such as impairment in axonal transport and synaptic function, oxidative stress, dysfunction of lysosomes and mitochondria [25,26], activation of microglial cells and protein aggregation, also contribute to neuronal damage [27]. Other factors such as genetics, age, and environmental factors influence the disruption of neuronal homeostasis and aggravate the existing neurodegeneration by activating the signaling of different molecules, ultimately causing cell death and declined functioning of the nervous system [28].

With the advancement of technology in recent years, the understanding of pathology and genetic changes invoked in neurodegenerative diseases has significantly improved but is still unsatisfying. Due to complex biology, the connection between the origin and execution of the death of neurons is still lucid [2]. The pathway involved in cell death and the mechanism responsible for its activation is still under question, and unraveling it is important to drive the development of new target-oriented therapeutic medications. There are several pathways that regulate the cell death of neurons, which are explained below.

### 2.1. Exhibition of Necroptosis, Apoptosis, and Related Processes in Neuronal Death

Apoptosis can be defined as a type of programmed cell death that is mainly associated with the disintegration of deoxy ribonucleic acid (DNA) and the destruction of several nuclear and cytoskeletal proteins, which leads to immune cell-mediated phagocytosis. The protein caspases execute a chain of proteolysis, which ultimately results in cell death [29]. During the development of the central nervous system, excess neurons are removed by neuronal apoptosis, followed by acute insults that range from excited conditions to injuries [30]. The activation of caspase 8 by extrinsic pathway and caspase 9 via intrinsic pathway leads to the activation of death receptors and mitochondrial damage, respectively, and eventually converges to caspase 3, which ultimately initiates downstreaming of neuronal apoptosis. It was established in a published paper that mice that lack caspase 3 expressions present excessive neuronal cells at birth and significantly reduced apoptosis [31]. The damaged mitochondrion in neurons initiates apoptosis with the help of proapoptotic protein Bax, belonging to the bcl-2 family, by activation of the mitochondrial membrane and release of cytochrome C and further caspase 9 activation [32]. This pathway mainly regulates apoptosis of the neurons even when the chief stimuli has not impaired the functioning of mitochondria. This indicates that the permeability of mitochondria broadly contributes to the apoptosis of neurons. It has been discovered that the neurons that lack Bax protein display no activation of caspases and are strongly protected from neurodegeneration even after prolonged insults [33].

Necroptosis is a form of programmed cell death that is independent of caspases and has been recently discovered to explain cellular characteristics of necrosis. Interferons, tumor necrosis factor, toll-like receptors, and signaling of other proteins and infections act as stimuli and induce necroptosis [34]. Apart from apoptosis and necroptosis, several other pathways that cause cell death have also been identified, such as iron-independent necrosis, known as ferroptosis; lysosomal cell death, known as auto lysis; and death by self-eating, known as autophagy. All these mechanisms are interrelated and cause neuronal cell death.

### 2.2. Cell Death Regulation in Response to Neuronal Stress

The signaling of the c-Jun amino terminal kinase (JNK) pathway induces traumatic responses that lead to the death of neurons. The role of the JNK signaling pathway has been characterized as the functioning of neurons, including responses that mediate neuronal injury [35]. Another protein, namely, dual leucine zipper kinase (DLK), regulates JNK signaling and upstream neuronal functioning. It is mainly triggered by neurotrophic growth factors, oxidative stress, injuries associated with neurons and axons, and misfolded proteins [36]. DLK proteins lead to JNK phosphorylation and translocate to the nucleus. This phosphorylation further leads to transcriptional stress and culminates in CNS apoptosis. The DLK proteins control the degeneration of neurons in the developmental phase, but if uncontrolled it progresses to conditions of injury in neurons and neurodegeneration [37].

The neuronal cells that lack signaling by DLK proteins fail to generate a normal transcriptional injury response and further attenuate the activation of caspases, and hence, are strongly protected from destruction upon exposure to stress [38]. The activity of protein kinase R-like endoplasmic reticulum kinase (PERK) can be hampered by the signaling of DLK proteins to prevent the phosphorylation and translation of eukaryotic initiation factor-2α in neurons post-acute injury [39]. The signaling of PERK is a component of unfolded protein responses and its activation by DLK proteins significantly demonstrates its participation in the destruction of neurons. Therefore, the signaling of proteins like DLK, JNK, and PERK induces stress responses that further lead to the signaling of pathways, leading to cell death [40].

### 2.3. Programmed Destruction of Neurons by the Degeneration of Axons

**Figure 2:**
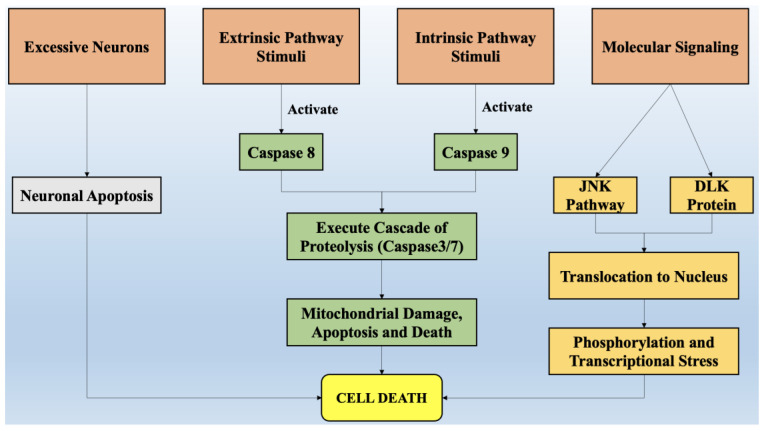
The factors that are involved in the pathways of cell death. Legend: JNK—c-Jun amino-terminal kinase; DLK—dual leucine zipper kinase.

The degeneration of axons can be mainly portrayed by forfeited connectivity in neurons [41]. This is the earliest clinical feature that has been discovered in most neurodegenerative diseases [42]. The degeneration of axons is a dynamic activity that depends upon the signaling of local axons and their transcriptional regulation within the body of neuron cells [43]. Axon degeneration, in conjunction with the apoptosis of neurons, further aggravates the condition. Several pathways such as the withdrawal of trophic factors, DLK/JNK signaling pathways [44], and several chemotherapeutic compounds appear to be involved in axonal degeneration, whereas the DLK pathway is considered to be the governing pathway that leads to axonal degeneration by activating the signaling of caspase proteins and ultimately neuronal cell death [45]. Figure 2 briefly mentions all the factors that are involved in the pathway of cell death via processes of neuronal apoptosis, the formation of a cascade of proteolysis that induces mitochondrial stress and damage, and the signaling of various proteins such as c-Jun amino terminal kinase (JNK) and dual leucine zipper kinase (DLK), which leads to translocation to the nucleus and ultimately causes cell death.

Apart from the mechanisms stated above, there are several other factors that lead to neuronal cell death in diseases, such as protein homeostasis, [20] mitochondrial dysfunction, neuroinflammation, selective vulnerability, and linking of initiators and executioners of neurodegenerative diseases. All these factors run parallel and induce stress intracellularly in neurons, which leads to their death and chronic neurodegenerative disorders. These are also guided by accretion of the proteins that have misfolded or are defective in the endolysosomal system and oxidative stress.

The most common trait involved in the etiology of neurological disorders like Alzheimer’s disease and Parkinson’s disease includes the aggregation of toxic misfolded proteins, which leads to synaptic dysfunction and the destruction of neurons [46] For instance, the extracellular β-amyloid plaques observed in Alzheimer’s disease in conjunction with intracellular neurofibrillary tangles formed by the hyperphosphorylation of the tau protein is the contributing factor in the pathophysiology of Alzheimer’s disease [47]. The precise mechanism that triggers the conversion of normal proteins into misfolded proteins is lucid; however, the misfolded proteins recruit healthy proteins into lethal oligomers and accelerate their aggregation. These aggregated proteins sequesters from their colony and prevent the functioning that is necessary for their survival [48].

During normal physiological conditions, proteins, with the help of endoplasmic reticulum chaperones, are folded into their native form while the misfolded proteins are engulfed by autophagosomes, lysosomes, and proteosomes, a process called protein homeostasis. In conditions of stress, the misfolded proteins are generated in bulk and their aggregation leads to toxic functioning due to loss of endogenous functioning, which drives neuron cell death. Protein homeostasis is a critical process that governs the overall health of neurons. Mitochondrial dysfunction and neuroinflammation also lead to cytotoxicity in the brain and play a major role in the pathophysiology of neurodegenerative diseases. These processes mainly include the release of cytotoxic inflammatory cytokines in the brain, which causes phagocytosis of damaged neurons [49].

A selective region in brain and subsets of neurons that are highly exposed to cytotoxic agents lead to disease progression, a phenomenon called selective vulnerability. For instance, the pyramidal neurons in the entorhinal cortex region of the brain are initially targeted in Alzheimer’s disease. Hence, different pathophysiology in different regions of the brain mediates neurodegeneration, and thorough knowledge of neuron dysfunction processes and cell death can open opportunities for the development of more targeted therapies and enhance the quality of life of the affected populations.

## 3. Oxidative Stress and Its Role in Neurodegeneration

The most vital entity for all living organisms is oxygen. Oxygen plays an important role in the physiological functioning of the body, as it is involved in several processes, such as tissue formation, and is a basic element for the growth of every cell. It is highly crucial in inducing gene transcription and signal transduction [50]. However, in excess it may produce detrimental outcomes. The conditions of stress occur mainly due to an imbalance between concentrations of reactive oxygen species (hydroxyl free radical, oxygen) and antioxidants [51]. The imbalance arises mainly due to two factors, which include the excessive synthesis of reactive oxygen species or a disturbance in the antioxidant system of the body [52]. Mitochondria supplies adenosine triphosphate (ATP) to the cells by breaking down glucose molecules, a process called oxidative phosphorylation, and by the synthesis of several other essential biological molecules. The proteins and enzymes required in oxidative phosphorylation are mainly programmed by the DNA of the mitochondria. Apart from the production of ATP via electron transport chain and oxidative phosphorylation, mitochondria are also involved in producing molecules that have a tendency to overcome oxidative stress via apoptotic mechanisms and other functions in cells [53].

The enrichment of mitochondria with enzymes involved in redox reaction and dysfunction of mitochondria is the principal cause of oxidative stress and excessive production of reactive oxygen species in the cellular environment. A phospholipid called cardiolipin is found in the membrane of mitochondria and is specifically involved with proteins in the electron transport chain. Any abnormality in the oxidative phosphorylation process causes the dysfunction of mitochondria cells and the generation of reactive oxygen species (ROS) made up of univalent oxygen molecules. The most important molecule in reactive oxygen species is nitric oxide, which regulates the production and relaxation of the muscle cells of vasculature, leukocyte adhesion, platelet aggregation, angiogenesis, thrombosis, hemodynamics, vascular tone, and many more [54].

The major sources of free radicals include redox metabolism in mitochondria, the metabolism of phospholipids, and several other proteolytic pathways. For normal physiological functioning of the body, the concentration of reactive oxygen species must be low. High concentrations and excessive exposure to ROS leads to the destruction of macromolecules such as DNA, proteins, and lipids, which ultimately cause necrosis and apoptotic cell death [55].

Oxidative stress is also the key element that regulates aging and several other neurological conditions. The chemical integrity of the brain controls the higher functioning of the central nervous system. The human brain is most susceptible to oxidative stress, as it devours a huge proportion of oxygen and is highly rich in lipids [56]. Higher oxygen consumption produces an abundant number of ROS. The membrane of the neurons is made up of polyunsaturated fatty acids, which are also prone to reactive oxygen species [57].

Several neurodegenerative diseases such as Alzheimer’s disease, amyotrophic lateral sclerosis, Huntington disease, and Parkinson’s disease are a result of alterations in biochemical and biomolecular components mainly due to oxidative stress. Hydrogen peroxide, a highly reactive hydroxyl radical and super oxide anion, are some of the negative oxygen species that are involved in neurodegeneration. Therefore, a dire need arises to understand the principal role of oxidative stress in the etiology of neurodegeneration. Apart from reactive oxygen species, reactive nitrogen species such as nitric oxide also possesses destructive effects on neurons [58]. It is important to understand the pathophysiology involved in cell death via ROS and initiate treatment by targeting the specific pathways involved to combat these diseases.

**Figure 3:**
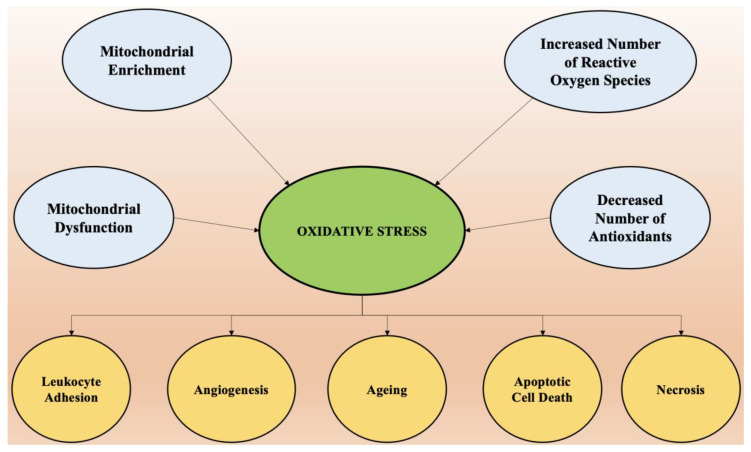
The factors that cause oxidative stress and the physiological changes that come as a result of increased reactive oxygen species.

Oxidative stress is the predominant factor responsible for the pathogenesis of numerous chronic diseases such as diabetes, rheumatoid arthritis, obesity, etc., and almost all neurological disorders. Scavenging of free radicals by antioxidants can prevent aging and oxidative stress-mediated pathological conditions. The factors that cause oxidative stress and the physiological changes that come as a result of increased reactive oxygen species are presented in Figure 3.

An insufficiency in the antioxidant-medicated defense system leads to increased concentrations of oxidative species, which is highly problematic, as neuron cells are made up of polyunsaturated lipids, which are extremely susceptible to oxidation and create deleterious effects on biomolecules [59]. Changes in proteins, lipids, enzymes, DNA, ribonucleic acid (RNA), and other biomolecules under stress conditions can be used as biomarkers due to their highly sensitive nature and thus can be helpful in the determination of oxidative stress [60]. Reactive oxygen species such as OH, ONOO, etc. can be generated by altering the functioning of proteins and lipids.

The heterocyclic bases of DNA and RNA, mainly guanine, is extremely prone to oxidative damage and can be attacked by reactive oxygen species, which leads to the formation of 8-hydroxy-2-deoxyguanosine and 8-hydroxyguanine. These modified bases, when increased to a certain extent in the brain, produce significant damage and are the principle cause of Parkinson’s disease [61]. The carbonylation and nitration of proteins is also predominantly witnessed in brains of Alzheimer’s patients.

Lipids form the plasma membrane of the neuron cells and act as a barrier between extracellular and intracellular spaces, and hence play a major role in the functioning of neuron cells [62]. However, these lipids are easily attacked by free radicals and go through lipid peroxidation, which decreases the fluidity of the membrane and causes leakage, which encourages the entry of substances that are usually not allowed to pass intracellularly but now cross these barriers through specific channels, thereby mutilating enzymes, membrane proteins, and receptors [63].

A significant role of pro-oxidants in neurodegenerative disorders has also been established, apart from the endogenous and exogenous sources of oxidative stress. Rich sources of polyphenols such as vegetables and fruits play an important part in generating defense antioxidant mechanisms. Awareness to increase such ingredients in the diet on a routine basis can be highly beneficial. However, the occurrence of neurodegenerative diseases and other chronic health problems is not solely dependent upon antioxidants, and there may be several other factors that induce oxidative stress and have been identified as pro-oxidants [64]. Pro-oxidants are any xenobiotic or endobiotic species that increases the production of reactive oxygen species or reactive nitrogen species by inhibiting the antioxidant system and triggering oxidative stress in tissues and cells. Exogeneous prooxidants include dietary supplements, pathogens, and toxicant drugs, whereas endogenous prooxidants include environmental pollution, metabolites of drugs, anxiety, climate change, and many more.

Heavy metals also play an important role in oxidative stress and have harmful effects on the reproductive system, respiratory system, central nervous system, cardiovascular system, and vital organs of the body [65]. These metal ions accumulate in the environment of the human body and combine with other xenobiotics and exert deteriorating effects on the immune system and hematology. Exposure to heavy metal ions such as lead and mercury and a deficiency in necessary metal ions like zinc and selenium lead to an increase in oxidative stress and produce deleterious effects by generating abnormality in the redox functioning of the cell [66].

The role of mercury in biological processes is minimal but its presence and accumulation in the body can produce harmful effects. Oxidative stress, which is mediated by the presence of mercury, can lead to the damage of biomolecular membranes, which further initiates the synthesis of hydrogen peroxide [67]. The neurotoxic effects of mercury have been discovered, and it has been determined that excessive mercury causes tremors and memory issues in the brain, accompanied by altered hearing and vision. Another heavy metal, lead, is also toxic to humans because of its nature to induce oxidative stress [68]. The damage produced by lead significantly depends upon the dose, route and duration of exposure, health status, and age of the individual. Lead has a high affinity for the -SH group, which is mainly present in amino acids and other metal cofactors that cause a significant reduction in the enzymes responsible for antioxidant activity, thereby elevating oxidative stress and causing failure of vital organs.

Another heavy metal, arsenic, is also prone to binding with the -SH group of glutathione, leading to the production of hydrogen peroxide. Arsenic is also responsible for the inhibition of glucose absorption in cells and the oxidation of fatty acids [69].

Recently, the role of ferroptosis has also emerged as being involved in inducing oxidative stress, which causes neuron cell damage and cell apoptosis [70,71]. The process of ferroptosis is mainly dependent upon the production of soluble and lipid reactive oxygen species by enzymatic reactions that mediate the transitioning of iron metal. Ferroptosis-mediated oxidative stress and mitochondrial dysfunction causes the death of neurons as well as cell apoptosis and targeting them can be an efficient strategy to prevent neurodegenerative diseases [72].

### 3.1. Mechanism of Oxidative Stress

Reactive oxygen species are mainly produced by a dysfunction in the mitochondria. The range of dysfunction through mitochondria is very wide and comprises a decrease in ATP production, altered regulation of calcium, brain iron accumulation, respiratory chain dysfunction, perturbation in the dynamics of mitochondria, and deregulation of mitochondria clearance [73].

In patients with Parkinson’s disease, several genes have been identified that strongly confirm the association of mitochondrial function with oxidative stress in the pathogenesis of the disease. The loss in functioning of the DJ-1 gene causes oxidative stress and exerts a neuroprotective role in response to its antioxidant characteristic in mitochondria [74]. Mutations in the recessive form of the PINK1 gene leads to the onset of Parkinson’s disease. PINK1 is a mitochondria kinase that regulates cytosolic calcium, and deficiency in this gene leads to impairment of calcium influx in mitochondria and causes mitochondrial calcium overload, which leads to the opening of the mitochondrial permeability transition pore (MPTP) and permits the translocation of proapoptotic molecules to cytosol from mitochondria [75].

Apart from Parkinson’s disease, the dysfunction of mitochondria has also been identified in the pathology of Alzheimer’s disease and other common neurodegenerative disorders. The ROS are produced via the oxidation of nicotinamide adenine dinucleotide phosphate (NADPH) and selective neural degeneration. The crucial role of NADPH oxidase in neurodegenerative disease has been confirmed in several animal models. The active form of NADPH oxidase relocates proteins to membranes to enhance healthy neuronal activity and requires the breaching of ion/anion channels to compensate the charge. In microglial cells activated by beta-amyloid proteins, the superoxide production is inhibited [76]. The stimulation of NADPH oxidase by beta-amyloid proteins in combination with the abundant synthesis of nitric oxide can cause the destruction of the surrounding cells. The beta-amyloid proteins also activate NADPH oxidase by causing the entry of calcium into the astrocytes, thereby preventing their entry into the neuronal cells. After NADPH oxidase actuation [77], the mitochondrial membrane is depolarized, which, combined with calcium, leads to the opening of MPTP and changes the membrane structure by phospholipase C stimulation. This signal of oxidative stress is further carried to neighboring neuronal cells that are highly exposed compared to astrocytes. [78]. When the neurons have multiple synapses and long axons, they have high energy requirements for transportation through axons and long-term plasticity.

To fulfil such big energy requirements, a high demand for ATP is rendered, thereby increasing levels of reactive oxygen species in conditions of stress, which imparts a higher degree of neurodegeneration. For example, the neurons in the hippocampus region of the brain generate greater levels of superoxide anions and comprise increased expression of antioxidant and reactive oxygen species that produce genes [79]. The amplified oxidative stress in intrinsic surroundings leads to the dysfunction of mitochondria, which further generates more free radicals, thereby exaggerating the oxidative stress cycle.

**Figure 4:**
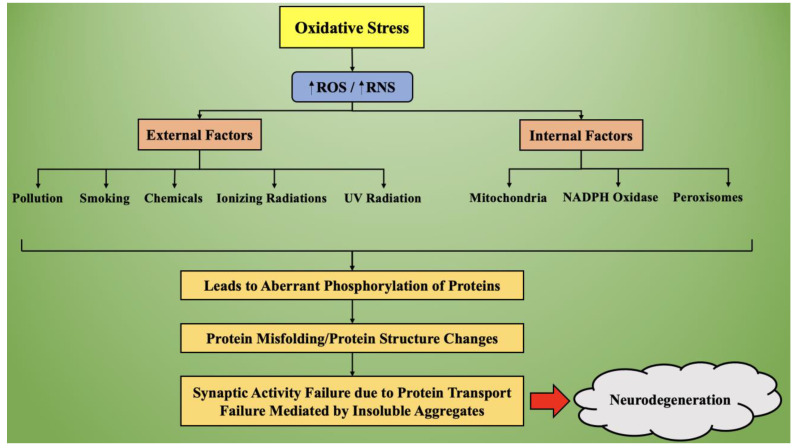
Synthesizing the process of oxidative stress-mediated neurodegeneration. Legend: ROS—reactive oxygen species; RNS—reactive nitrogen species; UV—ultraviolet; NADP—nicotinamide adenine dinucleotide.

Figure 4 represents the process of oxidative stress-mediated neurodegeneration. Based on external and internal factors, the production of reactive oxygen species increases in the body, which causes an overall increase in the oxidative stress. This oxidative stress causes aberrant phosphorylation of proteins, leading to synthesis of misfolded proteins, which alters the normal functioning of neuronal transmission. Due to altered protein structures, they get aggregated and lose the ability to transport, thereby hampering the synaptic activity and causing the initiation and progression of neurodegeneration.

### 3.2. Targeting of Oxidative Stress in Neurodegenerative Diseases

For the management of oxidative stress-mediated neurodegeneration, it is essential that antioxidant therapies be administered that target reactive oxygen species and suppress oxidative stress, thereby decreasing the intensity of neurodegeneration. Antioxidants are endogenous or exogenous molecules that antagonize the effects produced by reactive oxygen species and oxidative stress in the cellular system. These compounds neutralize the effect of reactive oxygen species and engulf any free radicals, thereby inhibiting conditions of oxidative stress [80].

By altering the foods in our diet, we can enhance the intake of antioxidants by consuming food products rich in flavonoids, lipoic acid, phenolic compounds, beta carotene, and ascorbic acid (Vitamin C). These naturally occurring antioxidants prevent the protein and lipid oxidation process in the body, thereby suppressing the generation of ROS and upstreaming the therapeutic barrier to oxidative stress. The ROS initiate excitotoxicity and modulate the glutamate receptors by over-activating them.

The drugs that target these receptors can be efficiently used in upstreaming the antioxidant profile and in the management of neurodegenerative diseases by blocking these specific receptors. For instance, Alzheimer’s disease can be slowed down by administering memantine, which targets N-methyl-D-aspartate receptor and imparts modest benefits to the patient suffering from a moderate to severe range of the disease. An upcoming therapeutic aspect for the prevention of neuronal cell death and the regulation of oxidative stress includes vaccination of the individual against the potential toxic proteins that are formed in several types of neurodegenerative disorders. A promising example is vaccination against the beta-amyloid protein, which is mainly found in patients with Alzheimer’s disease. Vaccination against it prevents the formation of plaque and subsequent neuroinflammation.

## 4. Role of Neuroinflammation in the Onset of Neurodegeneration

Recent studies have demonstrated a bridge between chronic inflammation and neurodegeneration. Apoptosis or programmed cell death and necrosis lead to neuronal cell death in the brain [81]. An increased burden of neurodegenerative conditions on the health care system and a lack of effective treatments available pose an urgent need to identify new drug targets. The most common feature that has been found in several neurodegenerative diseases such as Parkinson’s disease and Alzheimer’s disease is chronic neuroinflammation. Glial cells have been identified as the mediators of neurodegeneration and are responsible for the onset and progression of these diseases.

Neuronal health is mainly monitored by the nervous system’s immune cells, called microglia [82]. These cells are activated upon any injury to neurons or infections, which further produces proinflammatory factors (M1 phenotype) or anti-inflammatory factors (M2 phenotype). For a healthy functioning of the human brain, it is necessary to have the correct balance between anti-inflammatory mediators, which allow for the repair and healing of tissues, and proinflammatory mediators, which clear the cellular debris and misfolded protein aggregation is maintained [83]. The activation of microglia cells in Alzheimer’s and Parkinson’s disease is mainly tilted towards the M1 phenotype, which leads to an exaggeration of inflammation and accelerates the progression of the disease [84]. The new therapies for the management of neurodegeneration include induction of the M2 phenotype and deactivation of the M1 phenotype in the brain.

Astrocytes are another type of glial cells that are present in the brain and regulate the maintenance and maturation of neuronal cells. They are highly sensitive and respond to injury very quickly, as do microglia cells [85]. Depending upon their activation, they can be neuroprotective by stimulating repair and reducing inflammation or be neurotoxic by promoting inflammation and contributing to the death of neuronal cells. Activated astrocytes also act as a proinflammatory factor in Parkinson’s disease and Alzheimer’s disease [86]. Their role has been implicated in the breakdown of the blood–brain barrier, thereby encouraging the infiltration of immune cells into the brain, which increases neuronal death by excessive stimulation and impairment of the uptake of neurotransmitter glutamate. Another type of glial cell, called oligodendrocytes, also poses a significant role in neurodegeneration [87]. Oligodendrocytes form a sheath of myelin around the nerve fibers, which permits the efficient and rapid transmission of electrical impulses across neurons and thus induces signal transmission. Damage in these cells has been attributed to the progression of multiple sclerosis and other neurodegenerative diseases in which the immune system attacks oligodendrocytes and damages the myelin sheath, thereby reducing levels of myelin proteins in brain tissue [88].

### 4.1. Sources of Neuroinflammation

The mechanisms involved in neuroinflammation by biomolecular or cellular pathways are alike in aging and metabolic diseases such as depression, dementia, diabetes, and hypertension, as well as cerebral insult conditions such as stroke, and contribute silently to neuroinflammation. In elderly groups of patients, the mechanism of inflammation has been strongly associated with the pathogenesis of dementia and functional impairment [89]. Local and systemic inflammation in CNS significantly contributes to the development of small cerebral vessel diseases such as vascular dementia, which is mainly hypothesized as microvascular changes that cause a state of chronic hyper perfusion and lead to the death of oligodendrocytes and consecutively cause the destruction of myelin fibers, which increases inflammation in the affected brain region [90,91].

Other markers of inflammation such as C-reactive protein (CRP) can help with the prediction of clinical and subclinical atherosclerosis and play a major role in the identification of brain hemorrhage and conditions of stroke. Various other metabolic disorders such as diabetes [92], obesity, and hypertension also lead to dysfunction of the adipose tissue and induce low-grade inflammation, thereby worsening neuroinflammation in patients with a history of stroke [93]. Another factor that induces the synthesis of proinflammatory cytokines is aging processes. The inflammation triggered due to aging in the brain manifests chronic activation of parenchymal and perivascular macrophages and increases expression of microglial cells, thereby increasing the number of astrocytes. This chronic activation and signaling of pro-inflammatory cytokines increases the vulnerability of the patient to psychiatric disorders [94].

Females that are obese are mainly observed to have elevated concentrations of interleukin-6, CRP, and adipokines [95]. All these pro-inflammatory cytokines contribute positively to manifesting symptoms of depression and anxiety. Upon surgical removal of fat tissues, an alleviation in anxiety was noted. Further metabolic diseases such as hypertension and obesity accompanied with older age groups pose as a prevalent risk factor in the initiation of cognitive dysfunction, including depression, dementia, and other neurodegenerative disorders [96].

The biological mechanism responsible for depression is still lucid, and patients are administered conventional anti-depressant therapy for the correction of the disease; however, it has been noted that neuroinflammation suppresses the beneficial effects of the therapy in one-third of patients, leading to resistance in treatment [97]. The putative mechanism that links inflammation and neurodegeneration includes elevated pro-inflammatory cytokines, mainly IL-6 or IL-8 [98], oxidative stress, higher glutamate, and uncoupling of endothelial nitric oxide synthase. In severe psychiatric illnesses such as major depressive disorder, evidence of neurovascular dysfunction was determined to be indirectly associated with increased concentrations of inflammatory cytokines in the periphery [99].

Hyperactivated immune response results in an inflammatory cytokine rush and poses problems to the central nervous system [100]. Immune responses are generally triggered upon exposure to certain infectious agents such as viruses, bacteria, or any other associated pathogens [101]. A pathogen can enter the CNS through two different hypothetical pathways, including hematogenous dissemination, through which the pathogen gains direct access to the brain via the blood–brain barrier, and neuronal retrograde dissemination [102,103]. Different pathogens interact in distinct ways and lead to the activation of macrophages and further stimulation of inflammatory cytokines and neuroinflammation.

### 4.2. Neuroinflammatory Process

Through emerging evidence, the role of neuroinflammation in neurological diseases has been well documented, and by controlling the key factors that are responsible for the immune and inflammation processes, can serve as a key to the prevention and delay of most late-onset CNS disorders [104]. Poor quality of life and unhealthy food habits have led to the onset and progression of several diseases such as stroke, hypertension, diabetes, obesity, etc., which causes alteration in lipid hormones, adipokines, and inflammatory cytokines, thereby inducing adverse regulatory responses [105]. These metabolic disorders also regulate chronic activation of the pro-inflammatory cytokines even in the CNS, which makes the population vulnerable to neurological disorders.

A correlation between neurological disorders such as anxiety and depression and proinflammatory cytokines has been developed, which indicates their chief role in the instigation of cognitive dysfunction, thereby favoring neurodegradation. An increase in the level of peripheral inflammatory markers such as interleukin (IL)-6 and IL-8 is associated with mortality from suicide and depression. The complement cascade of the activation of microglial cells in response to pruning synapses is a biological mechanism that commonly occurs in neuroinflammatory processes and the development of a healthy brain. The generation of neurotrophic factors can be compromised by the activation of the immune system and lead to microglial cell-mediated secretion of cytotoxic factors.

Microglial cells are the main agents involved in neuroinflammation, with the second being astroglia cells [106]. Astrocytes are responsive to all forms of CNS insults due to the reactive astrogliosis process. The activation of astrocytes is highly complex, multistage, and a pathogen-specific reaction, and mainly aims for neuroprotection and recovering the injured and damaged neuronal tissue. It is evident that the sustained inflammatory responses in the central nervous system support the major contribution of astrocytes and microglia to neurological disease progression, thereby implying their chief role as effectors of neuroinflammation in neuron dysfunction and cell death [107]. Endothelial cells, neurons, and many other cells act as receptors for cytokines and inflammatory mediators, thereby activating the signaling of inflammatory responses in the brain.

### 4.3. Neuroinflammation Mediated by Microglial Cells

Microglial cells are widely distributed throughout the region of the brain and spinal cord and are mainly present in the substantia nigra and hippocampus region of the brain. These cells account for 5–20% of the population of all glia cells in the central nervous system and chiefly represent the immune response, as they possess the ability to perform phagocytosis, manifest the secretion of cytotoxic factors, and act like antigen-presenting cells. These cells originate in the primitive yolk sac during hematopoiesis and later rise and migrate to the developing neural tube [108]. These cells comprise the major cellular component of the immune system of the brain. In healthy conditions, microglia cells initiate quick responses to infection and several other stimuli, thereby modulating inflammation and protecting the environment of the brain.

Microglial cells sense different types of stimuli such as bacterial, viral, or fungal infections; antibodies; compliment factors; cytokines; and chemokines that threaten the homeostasis of the CNS and become activated [109]. These cells comprise two main functions: immune defense, by acting as sentinels and detecting the first signs of pathogen invasion and tissue damage; and maintenance by remodeling, repairing, and supporting the tissues of the central nervous system. In response to environmental and toxic factors, microglial cells induce inflammatory and immune responses in the CNS by becoming functionally polarized and expressing specific functioning reactions by activating pro-inflammatory cytokines and expressing the surface of receptors through the release of numerous soluble factors [110]. The stimulus can polarize microglial cells toward the distinctive functional roles, which causes the expression of different proteins and cytokines.

Activated inflammatory phenotype microglial cells upregulate the release of proinflammatory cytokines such as IL-1β, IL-6, IL-12, IL-23, inducible nitric oxide synthase (iNOS), and tumor necrosis factor (TNF- α), whereas activated anti-inflammatory phenotype microglial cells lead to the upregulation of arginase-1 [111], insulin-like growth factor (IGF-1), mannose receptor (CD206), chitinase 3, and triggering receptor expressed on myeloid cells 2 (TREM2). All of these inflammatory factors contribute to the process of microglial cell activation, which further leads to the production of cytokines and other inflammatory mediators, hence contributing to apoptotic cell death and neurodegeneration.

### 4.4. Neuroinflammation Mediated by Astrocyte Cells

These cells are heterogeneous in type and are highly abundant in the CNS [112]. Based on the stage of development, localization, and subtype, the morphology of the cells can change. For instance, the astrocytes in white matter are fibrous and exhibit long unbranched processes, whereas the astrocytes in grey matter are protoplasmic and possess short branches. These cells act as sensitive markers for the detection of any disease in the neuronal tissue and are responsible for the crucial functioning of the central nervous system [113]. They play a primary role in the transmission of signals in the synapses and control neural circuits. Astrocytes control the CNS environment by maintaining ion homeostasis, blood flow, and pH regulation and controlling oxidative stress. Based on the activities performed by astrocytes, these cells, in combination with microglial cells, act as the main factor in neuroinflammation [114]. In addition, astrocytes rapidly detect damage signals after any injury and undergo changes in their functioning and morphology in response to control and remove brain insults, but if not controlled, the response may have deleterious consequences.

The pathway that leads to the activation of astrocytes is still lucid and several factors that trigger the activation of these cells are involved in the neuropathology of neurodegenerative diseases [104]. It has been demonstrated that in patients with Alzheimer’s disease, astrocytes are activated by the presence of amyloid proteins, thereby causing local inflammation. The internal activation of astrocytes causes the transcription of nuclear factor kappa B (NF-ҡB), which further regulates the secretion and adhesion of chemokines and causes the infiltration of lymphocytes in the cerebrospinal fluid, thereby increasing inflammatory reactions and leading to neurodegeneration [115]. Blocking the activity of NF-ҡB protein in astrocytes can significantly reduce neuroinflammation, and it is suggested as a potential therapy for Alzheimer’s disease and several other associated neurological disorders.

**Figure 5:**
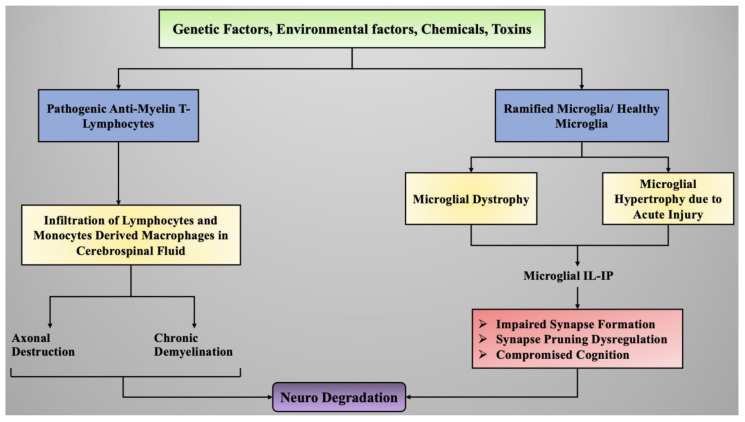
Environmental factors, toxins, chemicals, or genetic factors that can lead to the activation of anti-myelin T-lymphocytes. Legend: IL—interleukin; IP—inducible protein.

Figure 5 depicts the exposure to certain environmental toxins, chemicals, or genetic factors that can lead to the activation of anti-myelin T-lymphocytes. These T-lymphocytes, upon activation, initiate immune responses and inflammation as defense factors. Prolonged exposure to these toxins can induce increased production of inflammatory mediators and cytokines and generate hyper-immune response, which causes the infiltration of inflammatory cytokines and lymphocytes into the cerebrospinal fluid and initiates neuroinflammation. Microglial cells (plural: microglia) are the chief neuronal cells responsible for immune responses and inflammation in the central nervous system. Exposure to certain toxicants can induce alterations in healthy microglial cells, leading to their hypertrophy and dystrophy based on the intensity of the stress and thereby causing neuroinflammation and ultimately neurodegradation.

### 4.5. Strategies to Fight off Neuroinflammation in Neurodegenerative Diseases

With progressive understanding of the pathophysiology of neurogenerative diseases, the role of neuroinflammation in the progression of neurodegenerative diseases is highly acknowledged. Most of the pathologies of CNS are characterized by an early activation of microglial cells, which further initiate an early acute reparative phase via the effective removal of threatening compounds and toxic agents. However, this response mostly fails to completely remove all the threats and results in a vicious cycle of unresolved cytotoxic inflammation [116]. This has led to the development of therapies that target arresting such vicious cycles by resolving immune responses and recruiting systemic monocyte-derived macrophages. Below are some of the ways that can aid in fighting neuroinflammation and prevent neurodegenerative diseases [117]. The recruitment of CNS-specific T-cells, myeloid cells, microglial cells, and CNS-infiltrating monocyte-derived macrophages can be used in neuroprotection and to prevent neuroinflammation. Circulating myeloid cells possess neuroprotective effects and support CNS during conditions of neuroinflammation and neurodegradation [118]. It has also been observed that infiltrating blood-derived macrophages exhibit the capacity for phagocytosis and possess more neurotropic and anti-inflammatory effects than microglial cells.

## 5. Conclusions

The number of patients suffering from neurological disorders is increasing at an alarming rate. Several pathologies have been identified that cause the onset of neurological diseases; however, there is still a significant gap in the understanding and targeting of them for the development of medications for prevention and cure. With advancing technologies, the understanding of molecular mechanisms in populations exposed to vulnerable neurodegenerative diseases is also improving, which will lead to deeper insights into the functional interplay between several factors such as oxidative stress, neuronal cell death, and neuroinflammation in the occurrence of neurological diseases, which has been described in the article. Eventually, studying pathological mechanisms can lead to the targeted derived production of therapies that will improve global health and invoke management of neurological disorders, thereby improving the quality of life of individuals.

## Figures and Tables

**Table 1:** Types of neurodegenerative diseases according to the brain region affected.

Brain Region Affected	Types of Neurodegenerative Diseases
Basal ganglia	Parkinson’s disease
	Huntington disease
	Alzheimer’s disease
	Frontotemporal degeneration
Thalamus	Alzheimer’s disease
	Frontotemporal degeneration
	Multiple sclerosis
Cerebellum	Multiple sclerosis
	Multiple systemic atrophy dystonia
	Alzheimer’s disease
	Spinocerebellar ataxia
Cerebral cortex	Frontotemporal dementia
	Alzheimer’s disease
	Tremors
	Parkinson’s disease
	Huntington disease
	Amyotrophic lateral sclerosis
	Neuro psychiatric disorders
Brain stem	Frontotemporal lobar degeneration
	Parkinson’s disease
	Huntington disease
	Frontotemporal dementia
	Amyotrophic lateral sclerosis
	Spinocerebellar ataxia
